# A Review on the Role of Wollastonite Biomaterial in Bone Tissue Engineering

**DOI:** 10.1155/2022/4996530

**Published:** 2022-12-13

**Authors:** Chirotaw Getem Zenebe

**Affiliations:** Department of Chemical Engineering, Kombolcha Institute of Technology, Wollo University, P.O. Box: 208, Kombolcha, Ethiopia

## Abstract

Millions of people around the world have bone-tissue defects. Autologous and allogeneic bone grafting are frequent therapeutic techniques; however, none has produced the best therapeutic results. This has inspired researchers to investigate novel bone-regeneration technologies. In recent years, the development of bone tissue engineering (BTE) scaffolds has been at the forefront of this discipline. Due to their limitless supply and lack of disease transmission, engineered bone tissue has been advanced for the repair and reconstruction of bone deformities. Bone tissue is a highly vascularized, dynamic tissue that constantly remodels during an individual's lifetime. Bone tissue engineering is aimed at stimulating the creation of new, functional bone by combining biomaterials, cells, and factor treatment synergistically. This article provides a review of wollastonite's biomaterial application in bone tissue engineering. This work includes an explanation of wollastonite minerals including mining, raw materials for the synthesis of artificial wollastonite with various methods, its biocompatibility, and biomedical applications. Future perspectives are also addressed, along with topics like bone tissue engineering, the qualities optimal bone scaffolds must have, and the way a scaffold is designed can have a big impact on how the body reacts.

## 1. Introduction

The first step in creating an effective scaffold for bone healing is to comprehend the physiology of real bone. Bone is a remarkable organ that plays a significant role in vital physiological processes in humans, including blood production, mineral storage and homeostasis, blood pH management, and various progenitor cells (mesenchymal and hemopoietic) housing [1, 2]. It mostly consists of cancellous (trabecular) and cortical bone. The cortical tissue is the outermost and denser boundary of the bone mass, whereas the cancellous tissue makes up the interior, softer, lighter-density, and highly vascularized core of the bone mass (50-90 v/v%) [3, 4].

The cancellous bone is exceedingly porous (30-90%) [5, 6] and is wherein maximum metabolic activities take place. It has a compressive strength of 7-10 MPa and is characterized by a lower elastic modulus and very low tensile strength (Y < 2 MPa) [7, 8]. Cellular activity affects how permeable the cancellate tissue is, and mediators that impact cellular activity can change the trabeculate structures that determine pore size. It allows the passage of nutrients and metabolic waste and house bone marrow, blood vessels, and many other biological components [8, 9]. The cortical tissue has a higher elastic modulus, higher stiffness, and lower toughness due to its increased mineralization content and lack of organic matter [10]. It serves as a protective cage for the interior delicate cancellous tissue and has a denser, tougher structure (5-30% porosity). Because of its high compressive strength range of 130 to 225 MPa, cortical bone also serves as the primary structural support element in the body (Y: 60-160 MPa; E: 3-30 GPa) [11]. The combination of these two tissues gives bone a special mechanical structure that can withstand more loads and deformations than each of its constituent parts could have done on their own [12].

The calcium phosphate mineral family member nanohydroxyapatite (HA, Ca_10_(PO_4_)_6_(OH_2_)), which makes up about 70% of bone, is a typical complex tissue with a hierarchical structure [13]. The remaining 20-30% is made up of a mix of water, which is connected to the collagen protein family, and other proteins and proteoglycans, which make up the organic components of bone mass [14]. Volume and weight fraction ratios of hydroxyapatite to collagen and water are not consistent. It depends on age and is also species-specific [2]. However, bone can lose one or all functions because of different damages.

Bone loss can take the form of actual faults and structural loss within an existing bone, as in osteopenia, or it can take the form of structural defects and areas lacking of bone due to external sources. Trauma and pathological conditions such as osteomyelitis, osteoarthritis, osteoporosis, and cancer can all result in a bone defect [14, 15]. Bone loss can occur primarily or secondarily. Primary bone loss can happen in bone illnesses like cancer. Metastatic illness is the most frequent cause of secondary bone loss. Osteoblastic or osteolytic characteristics may be present in tumor metastases. Both compromise the structure, nourishment, and metabolism of the bone [16, 17]. Trauma is the most common cause of bone defects. When the variety of bone defects surpasses the critical-size defect (CSD), the bone defects cannot heal with the aid of using themselves and require affordable medical intervention. Large bone defects or injuries are therefore severe issues in orthopedics that harm health and quality of life. These conditions can be brought on by old age, traffic accidents, fracture nonunion, and bone tumor removal [18–20].

An epidemiological study from 2010 to 2025 found that fractures increased in frequency in Europe at a rate of 28% per year, with an additional 25% economic burden, sparking intense interest in the field of bone repair medicine [21]. The quality of life of a patient is significantly impacted by bone loss. Consequently, the cost of bone-related medical operations is increasing. Broken or fractured bones are routinely among the most frequent traumatic injuries suffered by people, regardless of race, age, or gender. Due to this, with over two million surgeries performed each year, bone is now the second most frequently transplanted tissue worldwide [22, 23].

Autologous and allogeneic bone transplants are the main treatment options currently used in clinical settings to address large-sized bone defects. The fact that autogenous bone has good osteoinductivity, osteoconductivity, and osseointegration properties, which can form a coordinated structure and ensure mechanical strength at the bone-defect site, makes autologous bone grafts the gold standard for bone-tissue reparation and regeneration [24–26]. Autogenic bone transplantation does, however, have certain unavoidable downsides, including high prices, donor location neurovascular damage, inflammation, and infection. Allogeneic bone implants are frequently used to correct those flaws because they are readily available. However, issues with allograft bone transplantation include inadequate osseointegration, immunological denial, and the spread of blood diseases [27]. Researchers began looking for artificial substitutes for grafts made naturally from bone as a result of this circumstance. To replace autograft and allograft therapy, artificial bone has undergone several preparations. With the use of bone tissue engineering (BTE), it is possible to create artificial bone substitutes that have the same, better, or more functional properties as natural bone. BTE currently enables researchers to employ a variety of strategies to mix cells, biomaterials, and biological factors to create synthetic tissues for mending bone abnormalities [28, 29]. There are no obvious problems with this method, which has the advantages of great modifiability, low infectivity risk, and excellent biocompatibility [30, 31].

## 2. Bone Tissue Engineering (BTE)

BTE encompasses applying engineering techniques, biomaterial concepts, and finally the use of chemicals and growth hormones to enhance biological processes. It strives to successfully integrate bone regeneration at the locations of the host's defects without introducing any extra problems, such as donor site morbidity, immunogenicity, or poor vascularization. To create the ideal bioactive conditions and crucial mechanical support to encourage the formation of new bone tissue in defect areas, BTE uses biocompatible and biodegradable natural materials [32–34]. BTE induces new tissue repairing and regeneration by the synergy of cells, signals, and scaffolds [35]. Biomaterial-based scaffolds act as carriers for cells and messages. Scaffolding promotes cell adhesion, development, and differentiation in addition to providing a possibility for bone growth [36]. BTE attempts to create 3D constructs using a combination of cells and organic and synthetic materials and scaffolds that may be mechanically, structurally, and functionally superior to or functionally similar to the injured tissues [37, 38]. The implanted scaffold gradually deteriorates as the new tissue develops, eventually being totally replaced by it. Computer-aided design and computer-aided manufacturing (CAD/CAM) technology can be used to design and manufacture customized scaffolds. Typically, biological research should be done on the generated scaffolds [35, 39].

In vitro culture experiments, such as scaffold toxicity tests using animal or human cells, and in vivo animal experiments, such as repairing femur defects in rats, are two categories into which the fundamental strategies of biological studies can be divided as forecasting checks before the preclinical test [40, 41]. Animal models have been used in clinical investigations with successful outcomes. The optimal cell source, the type of biomedical treatment method, the preparation process, and the application of growth factors are just a few of the challenges that prevent laboratory-made biomedical devices and procedures from moving quickly to the clinical stage [42, 43]. The loading of medication, such as antibiotics, proteins, genes, anti-inflammatory medicines, and growth factors into scaffolds for tissue regeneration, is likely to have a role in the restoration of damaged tissues [44]. Wollastonite has lately emerged as a promising choice for biomaterials due to its beneficial characteristics, such as biocompatibility, biodegradability, nontoxicity, and excellent mechanical capabilities [45–47]. The porous wollastonite scaffold utilized in bone tissue regeneration may contain the desired medicines [48].

The single or multiphase scaffold composition, surface chemistry [49, 50], architectural parameters like pore size and interconnectivity [51–53], the rate of degradation and the degradation products [54], and the local mechanical properties of the matrix like modulus and viscoelasticity can all have a significant impact on the biological response. Surface alterations or the inclusion of bioactive components are commonly required for structural biomaterial scaffolds that provide appropriate mechanical properties to attain the desired combination of attributes [55, 56]. Host cells should be able to deposit extracellular matrix (ECM) and eventually replace the scaffold structure because scaffolds are not intended to be long-lasting implants [57, 58].

The architecture of the scaffold must be extremely porous and constant to permit cell and nutrient mobility [59, 60]. Optimizing the scaffold surface is also necessary to encourage cell adhesion, proliferation, and differentiation [61, 62]. The scaffold fabric needs to be versatile enough to be easily molded into a range of shapes and sizes to allow in situ therapy of particular patient bone defects. Successful material design for BTE requires an appropriate choice of biomimetic natural or tunable synthetic materials (biomaterials), including polymers, bioceramics, metals, and composites. This includes understanding the composition and structure of the local bone tissue [30, 58, 63].

**Figure 1:**
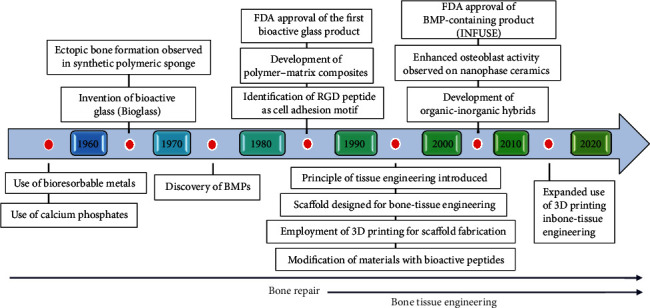
Timeline of the major milestone for BTE material design (reprinted with permission from [64]).

Figure 1 Shows the timeline of major milestones in biomaterials design for bone-tissue engineering. In the early stages of bone regeneration, calcium phosphates and bioresorbable metals were used. After that, research on bone formation in polymeric materials and the discovery of bioglass and the first man-made substance capable of attaching to living tissues were conducted. Further investigation led to the identification of bioactive compounds like proteins and peptides, and BTE was created as a distinct scientific field. Afterward, scaffolds were created using a variety of materials and then modified to trigger different biological processes. Regulatory bodies have evaluated products comprising different biomaterials as they were being developed for use in BTE applications, and some commercial products have been approved for use in clinical settings. BMP stands for bone morphogenetic protein; FDA stands for Food and Drug Administration of the United States; RGD stands for arginine-glycine-aspartic acid [64, 65].

A synthetic bone scaffold must meet the following biomimetic requirements: biocompatible, sufficient surface area with 3D structure [66], gives temporary mechanical support to the afflicted zone [67], serves as a substrate for osteoid deposition [68], vascularization, and bone in-growth that are made possible by their porous architecture [69, 70], elicits the migration of bone cells into the scaffold [71, 72], osteogenic differentiation support and promotion in the synthetic, nonosseous scaffold (osteoinduction) [69], promotes cellular activity for osseointegration, the process by which the scaffold is incorporated into the host tissue [69, 73, 74], to enable weight transfer to growing bone, deteriorate in a regulated manner [17, 75], provides harmless degradation byproducts [76–78], not incite an active chronic inflammatory response [79, 80], be capable of sterilization without loss of bioactivity [81, 82], and delivers bioactive molecules or drugs in a controlled way to accelerate healing and prevent pathology [83].

## 3. Synthesis, Structure, and Properties of Wollastonite

**Figure 2:**
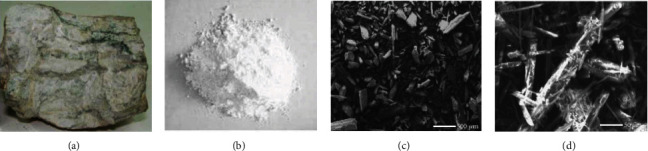
(a) Wollastonite ore. (b) Wollastonite powder. (c, d) SEM micrographs of coarse and fine wollastonite particles (reprinted with permission from [95]).

Wollastonite (CaSiO_3_) has a theoretical composition of 51.7% silicon dioxide (SiO_2_) and 48.3% calcium oxide (CaO) [84–86]. However, it might have trace or negligible levels of aluminum, iron, magnesium, manganese, potassium, sodium, or strontium [87] and manifests as large to acicular prismatic crystals that fracture [88]. Typically, it is white, but depending on its makeup, it could also be gray, brown, or red [89]. When impure limestones undergo metamorphosis (are heated and pressed), or when silica-bearing fluids are added to calcareous sediments during the metamorphic processes, wollastonite is created. Both times, calcite and silica react to form wollastonite and carbon dioxide [90]. Wollastonite naturally exists in two polymorphic states: the low-temperature phase *β*-wollastonite and the high-temperature phase *α*-pseudowollastonite [91, 92]. *β*-Wollastonite powders exhibit phase change and transform into *α*-wollastonite by sintering at temperatures above 1125°C [93]. The *β*-wollastonite mineral is obtained as a natural silicate mineral, whereas *α*-wollastonite is rarely found in nature [94]. The CaO-SiO_2_ binary phase diagram [93] shows that the wollastonite mineral phase is formed at lower temperatures than wollastonite. White, needle-like wollastonite mineral powder is its natural state. Micrographs taken using scanning electron microscopy show that wollastonite powder is solid and has rough surfaces as shown in Figure 2 [95–97].

**Figure 3:**
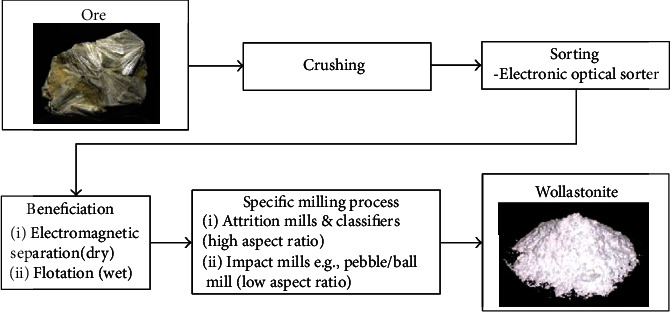
Production process of wollastonite powder from ore (reprinted with permission from [45]).

The size of wollastonite in ore form and the degree to which the shape and size are kept during processing determine the grades of wollastonite [45, 98]. Crushing, sorting, and beneficiation procedures are used to produce natural wollastonite powder. The grades of wollastonite particles will then be determined by specialized milling methods [45, 99] as shown in Figure 3. It is possible to produce low aspect ratio grades ranging from 3 : 1 to 5 : 1 and high aspect ratio grades with average lengths between 20 and 200 m using various mills and classifiers [45, 98]. Wollastonite's surface is typically modified using chemical coupling agents including titanate, silane, and betaine as well as surface modifiers like stearic acid and pimelic acid [100]. The physical properties of wollastonite are presented in Table 1.

**Figure 4:**
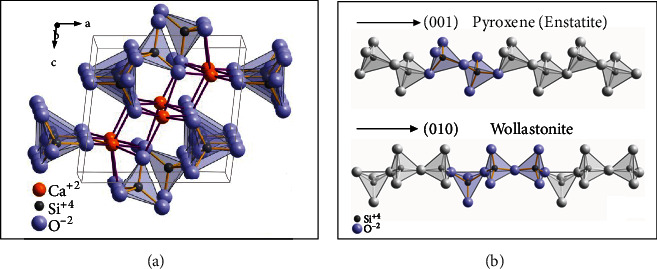
(a) Unit cell of triclinic wollastonite-1A and (b) tetrahedra arrangement within the chains in pyroxenes compared to wollastonite.

Wollastonite crystallizes triclinically in space group P1 with the lattice constants $a=7.94\;\overset{{}^{\circ}}{\text{A}}$, $b=7.32\overset{{}^{\circ}}{\text{A}}$, and $c=7.07\;\overset{{}^{\circ}}{A}$; *α* = 90, 03°, *β* = 95, 37°, *γ* = 103,43°, and 6 formula units per unit cell [105]. Due to its crystal structure, wollastonite belongs to the class of minerals known as pyroxenoids. It has been established that pyroxenoid chains are more kinked and have a greater repeat distance than pyroxene group chains. Infinite (SiO4) tetrahedra with shared vertices that are parallel to the *b*-axis make up wollastonite [106]. In pyroxenes, only two tetrahedra are required, as opposed to three in wollastonite, where the chain pattern repeats after each (Figure 4) [105].

China, Finland, India, Mexico, Canada, and the USA are the countries where most wollastonite minerals are located and mines [47]. The United States Geological Survey (USGS) reports that China would produce 890,000 tons of wollastonite in 2020, making it the world's top producer of the mineral. With 120,000 tons, India comes in second. Mexico and Canada are in third and fourth place with around 100,000 and 20,000 tons, respectively. There is a shortage of production data from USGS reports, but it is anticipated to be large and unchanged from 2019 [107].

Wollastonite is a tremendously interesting, but little-studied mineral that has a mixture of properties, such as a lack of volatile constituents [108], low shrinkage [109], fluxing characteristics [110], low dielectric constant and low dielectric loss [91, 111], thermal stability and low thermal expansion [98], low of loss on ignition [112] and low thermal conductivity [111], good bioactivity, biocompatibility, and degradability [113, 114], hence, is used in ceramic fabrication, medical material for artificial bones and dental roots, high-frequency insulator, filler for plastics and resins, paper, glazes for ceramics, metallurgy, paint, and frictional products [115, 116]. Due to its mechanical bioactivity and biocompatibility qualities, wollastonite, a glassy mineral with a calcium silicate base that is part of a class of bioactive and biocompatible materials, has a wider range of uses in medicine and as fillers for composite fabrication as well as for dental restoration and artificial bones [117–119]. Synthetic wollastonite can be prepared from various raw materials using different types of methods (Table 2).

## 4. Properties of Wollastonite Scaffolds and Implications to Use

When grafting native tissue, a bioactive material devoted to bone tissue restoration must encourage good bony growth [139, 140]. Biocompatibility and bacterial infections are the main challenges to current alternatives to bone transplants. Wollastonite-based scaffolds for bone tissue engineering have a greater potential to replace bone grafts in orthopedic applications due to their physicochemical characteristics, antibacterial properties, biocompatibility, and osteogenic induction effect on human bone marrow-derived stromal cells [141].

**Figure 5:**
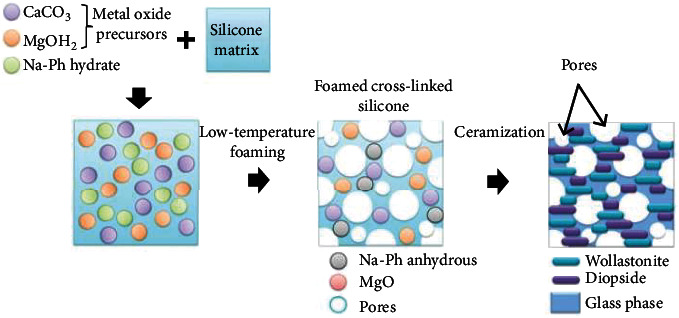
An illustration showing how sodium hydrate phosphate filler (Na-Ph hydrate) is used to create glass-ceramic foams from wollastonite-diopside (CaSiO_3_-CaMgSi_2_O_6_) polymer (reprinted with permission from [142]).

A more recent technology developed for bioactive glass-ceramic foams involves the use of polymer-derived ceramics [142–144]. In this approach, metal oxide precursors in the form of micro- or nanosized particles are added to a polymeric precursor (e.g., a silicone resin), allowing the production of silicate bioceramics (Figure 5). The foaming is obtained by water release from specific hydrated fillers. The foams are then sintered. Fiocco et al. showed the possibility of obtaining wollastonite-diopside (CaSiO_3_-CaMgSi_2_O_6_) foams with 77% porosity and compressive strength of 1.8 ± 0.3 MPa [145].

New materials for a bone replacement that offer extended implant lives, full integration, and suitable mechanical qualities were required on a clinical level. According to Dyson et al., human mesenchymal stem cells (MSCs) were utilized to fill porous apatite-wollastonite (A-W) glass-ceramic scaffolds created using the layer manufacturing procedure and selective laser sintering, to produce individualized bone replacements [146].

**Figure 6:**
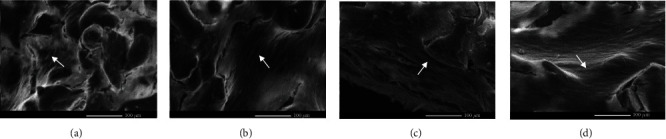
SEM examination of cell development on A-W scaffolds throughout time. After (a) 0 days, (b) 7 days, (c) 14 days, and (d) 21 days of culture in an osteogenic media, scaffolds were seeded with MSCs per scaffold and examined by SEM. Arrows indicate confluent sheets of cells (reprinted with permission from [146]).

The addition of 50% wollastonite to the hydroxyapatite matrix improves the porous scaffolds' strength, bioactivity, and biodegradability. The biodegradability tests reveal that the wollastonite-based composite scaffold could be quickly degraded when compared to pure hydroxyapatite [147, 148]. It was possible to develop biomedical applications for the *β*-wollastonite materials made from rice husk ash and limestone due to their favorable bioactivity and degradation characteristics. The SEM analysis of cell development on A-W scaffolds at various time points is displayed in Figure 6.

The loss of mass increased incrementally while the *β*-wollastonite samples were immersed in simulated body fluid (SBF) as a function of the soaking period [126]. According to the study of Ge et al., the wollastonite-hydroxyapatite composite biomaterial developed more blood vessels after 12 weeks of surgery. The material is safe in vivo therapeutically and can encourage the production and growth of new bone in the faulty location [147]. In earlier research, a porous glass-ceramic scaffold derived from apatite and wollastonite was produced with controlled pore size and porosity that revealed open macropores and met the fundamental criteria for a bone tissue engineering scaffold. The scaffolds also show great potential for use in bone regenerative medicine [149]. Wollastonite is an ideal candidate for biomaterial applications, particularly in orthopedics, as demonstrated by the development of the bioactive substance hydroxycarbonate apatite on the surface of a plasma-sprayed wollastonite coating soaked in simulated bodily fluid [150].

**Figure 7:**
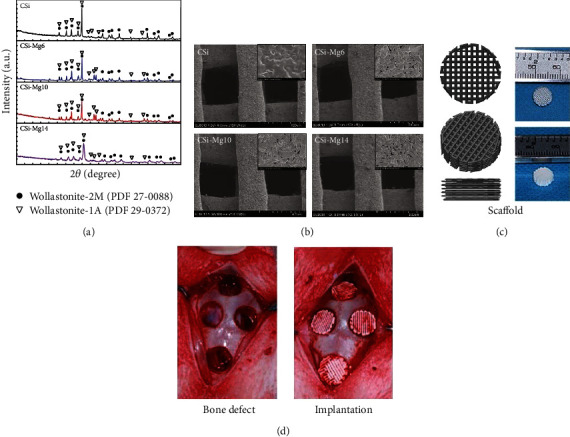
CSi-Mgx ceramic powders and scaffolds' characterization: (a) XRD patterns of ceramic powders, (b) SEM images of surface morphologies and microstructures of the ceramic scaffolds, (c) 3D model and macroscopic view of ceramic scaffold sample, and (d) bone defect and implantation of the ceramic scaffolds in rabbit skull defect (reprinted with the permission from [153]).

The Mg-doping wollastonite (CSi-Mg)/*β*-tricalcium phosphate (TCPx) scaffolds are capable of treating some challengeable bone defects, especially for load-bearing bone repair [151]. The two-step chemical precipitation and porogen burnout processes that were used to create the wollastonite/tricalcium phosphate macroporous nanosintered scaffolds resulted in less strength loss during the degrading phase. The scaffolds provide great potential for applications in bone reconstruction [152]. The CSi-Mgx ceramic powders and scaffolds' characterization are shown in Figure 7.

Xie et al. achieved ultrahigh strength bioceramic porous (>120 MPa) scaffolds using dilute magnesium-doped wollastonite inks and 3D printing techniques [154]. They show exceptional strength and degradability on osteogenic capacity in rabbit calvarial defects, making the 3D-printed diluted magnesium doping wollastonite, CSi-Mgx scaffolds promising for bone regeneration in thin-wall bone defects [153]. The 3D-printed diluted magnesium doping wollastonite, CSi-Mgx scaffolds show amazing strength and degradability on osteogenic capacity in rabbit calvarial defects, making them promising for bone regeneration in thin-wall bone defects [155]. The morphological and physiologically identical properties of the bone scaffold are created using a combination of bioactive porous silicon and wollastonite. It can open up the door for treating particular orthopedic problems by altering the design using additive manufacturing. In order to build the scaffolds in various topologies, additive manufacturing using a selective laser melting technique has been used [156, 157]. Stereolithography and the lost-mold method with gel-casting were used to create highly porous ceramic scaffolds from a wollastonite glass power. These scaffolds' strength and modulus are equivalent to those reported for other porous ceramic scaffold materials of similar porosity created by various fabrication techniques [158, 159]. Silicon-wollastonite-based scaffolds produced by selective laser melting show high bioactivity and controlled growth of the hydroxyapatite-like layer on the surface of the structures [156].

Compression molding, heat processing, and salt particulate leaching method and strategy were used to successfully produce the composite scaffolds of poly(3-hydroxybutyrate-co-3-hydroxy valerate) (PHBV) with bioactive wollastonite [160]. One of the crucial factors for scaffolds designed for tissue engineering is water uptake [161]. This property influences the transport of water and nutrients into the scaffold, which promotes cell growth. The water absorption of the PHBV scaffold is altered when wollastonite (WOL) is added [162, 163]. The addition of 10 wt% wollastonite exhibited a different kinetic mechanism and absorbed 44.1% more water than the uncontaminated PHBV scaffold when compared to other samples. Therefore, PHBV/WOL scaffolds can be a good choice for biological applications [163]. He et al. prepared a scaffold with excellent mechanical properties and long-term stability using diopside- (DIO-) based porous bioceramic composites via dilute magnesium-substituted wollastonite reinforcing and three-dimensional (3D) printing. The scaffold can potentially be used in the clinic, especially for the treatment of osteonecrosis of the femoral head working as a bioceramic rod [164]. Schmidt et al. also demonstrated that the wollastonite-diopside glass-ceramic complex structures material may be used to make bioceramic scaffolds for bone tissue engineering. They maintained their structure flawlessly, showed no viscous flow, and uniformly shrank by around 25%. With Kelvin structures, the cell design has a compressive strength of more than 3 MPa at 83% porosity [165].

The use of three-dimensional (3D) bioactive glass-derived porous scaffolds is an excellent method for accelerating bone healing and regeneration in substantial osseous defect areas [166, 167]. By using the foam replica process and the aforementioned bioactive glass powders as the parent material, the macroporous glass-ceramic scaffolds were created from SiO_2_, P_2_O_5_, CaO, MgO, Na_2_O, and CaF_2_. The scaffold exhibits bioactive behavior in vitro while submerged in simulated bodily fluids, and the mechanical characteristics were also possibly acceptable to indicate use in load-bearing bone applications [166]. A possible technique for promoting mineralization while the scaffold is incubated in a simulated physiological fluid is the production of biomimetic paste-type inks produced from wollastonite and fish gelatin in a mass ratio similar to that of natural bone. Also highlighted are the bicomponent inks' capacity to create three-dimensional bioactive scaffolds and their anticipated osteogenic capabilities for applications involving bone regeneration [168].

## 5. Bone Tissue Engineering Applications of Wollastonite

Despite the high capacity of Wollastonite, little attention has been given to its application in the field of bone tissue engineering. In the following table (Table 3), different works published on wollastonite applications in bone tissue engineering are presented.

## 6. Conclusion

The goal of the paper is to review the applications of wollastonite in bone-tissue engineering by investigating the results presented in several published articles. It has been found that both natural and synthesized wollastonite can be a potential candidate for bone tissue engineering. However, the literature review indicated that there was still little data about the employment of wollastonite in bone tissue engineering and demonstrating further study would be performed, and that a lot of research would be needed about wollastonite, its composites, and its uses in bone tissue engineering. In conclusion, wollastonite illustrated different favorable properties; hence, it could be further investigated in diverse bone tissue engineering applications.

## Figures and Tables

**Table 1:** Physical properties of wollastonite [101–104].

Properties	Typical Value
Appearance	White
Shape	Acicular
Molecular weight (g/mol)	116.159
Specific gravity	2.87–3.09
Specific surface area (m^2^/kg)	845
Melting point (°C)	1540
pH	9.9
Water solubility (g/100 cc)	0.0095
Density (kg/m^3^)	2899.3
Mohs hardness	4.5 to 5.0
Coefficient of expansion (1/°C)	6.5 × 10^6^
Theoretical melting point (°C)	1540
Young's modulus (GPa)	303–530
Tensile strength (MPa)	2700–4100

**Table 2:** Raw materials and methods used to derive synthetic wollastonite with its main application.

Raw materials/precursors	Process	Synthesis temperature (°C)	Application	References
Eggshells and commercial silica	Microwave	1100	-	[93]
Eggshells and rice husk ash	Sol-gel	850	Biomedical	[113]
	Solid-state reaction	1000	Biomedical	[120]
Rice straw ash and calcium nitrate	Sol-gel	-	Biomedical	[121]
Silica sand and limestone	Solid-state reaction	1400	Biomedical	[3, 122]
Stone wastes and silica fumes	Solid-state reaction	1100	-	[123]
Rice husk ash and cement kiln dust	Solid-state reaction	1100	Ceramic	[124]
Zirconium oxychloride slag and CaO	Sol-gel	1000	Environmental	[125]
Rice husk ash and limestone	Microwave	950	Biomedical	[126]
	Sintering	1050	Ceramic	[127]
Eggshell and waste glass	Solid-state reaction	900	Ceramic	[128]
Calcium nitrate and sodium silicate	Coprecipitation	1200	Biomedical	[129]
Waste fluorescent glass and CaCO_3_	Sintering	1300	Environmental	[130]
Waste soda-lime-silica glasses	Sintering	1000	Ceramic	[119]
CaCO_3_ and sodium metasilicate-pentahydrate	Coprecipitation	1100	Biomedical	[131]
Bentonite clay	Sintering	850	Biomedical	[132]
Calcium nitrate and fumed silica	Solution combustion	950	Biomedical	[133]
Calcium nitrate and tetraethoxysilane	Sol-gel method	-	Biomedical	[134]
Ca(NO_3_)_2_ solutions and Na_2_SiO_3_ solution	Hydrothermal microemulsion	800	Biomedical	[46]
Limestone and SiO_2_ smoke	Solid-state reaction	-	Materials	[135]
Calcium chloride and sodium disilicate	-	1200	-	[136]
Silica and calcium carbonate	Hydrothermal	1000	-	[94]
NaSiO_3_ 9H_2_O and Ca(OH)_2_	Hydrothermal	800	-	[137]
Calcium nitrate tetrahydrate and colloidal silica	Combustion	600		[138]

**Table 3:** A summary of studies conducted on wollastonite applications in bone tissue engineering.

Application	Sample	Method	Reference
Implant/bone repair	Wollastonite/*β*-TCP porous ceramic scaffolds	Polymer sponge replication	[169]
Titanium (Ti) implant	Minerals (Mg^2+^ and Gd^3+^) biocompatible composite coating	Electrophoretic deposition (EPD)	[132]
Bone substitute application	Porous hydroxyapatite-wollastonite-reinforced alumina nanoparticles (ALN: 40-80 nm)	Space holder (SH) technique	[170]
Clinical applications/bioceramics	Wollastonite-containing glass-ceramic coatings on alumina	Airbrush spraying of glass-based aqueous suspensions followed by sintering	[171]
Antibacterial activity	Silver-doped wollastonite	Sol-gel method	[96]
Implant applications	Wollastonite (WA) glass-ceramic with titanium	Sintering	[172]
Osseointegration	Apatite-wollastonite/poly (lactic acid)	3D-printed polymer and ceramic macrostructures	[173]
Implant material	Wollastonite/titanium oxide nanofiber bioceramic composite	Sintering/electrospinning	[174]
Bone tissue engineering	3D wollastonite-diopside scaffolds	Direct ink writing of ink made of silicone polymer and inorganic fillers	[175]
Bioceramic scaffolds	Wollastonite-diopside scaffolds with tailorable shell micropores	Direct ink writing technique with coaxially aligned bi-nozzle system	[176]
Scaffolds for bone repair	Porous composite materials (poly(L-lactide) (PLLA) and apatite-wollastonite (AW))		[177]
Bioceramics	Wollastonite/gold nanoparticles (aunps)	Spark plasma sintering	[178, 179]
Bone tissue engineering	Hydroxyapatite (HA) and *β*-wollastonite (WT)	Coprecipitation method	[180]
Bioactive ceramic scaffolds	Mg-substituted wollastonite (Csi-Mg6) bioceramic powders	Wet-chemical co-precipitation	[181]
Ceramic composites for biomedical applications	Wollastonite/titanium oxide and hydroxyapatite	Spark plasma sintering	[182]
Biomedical applications; bone graft	Wollastonite/hydroxyapatite scaffolds	Polymeric sponge replica	[148]
Bone formation and growth	Wollastonite coating on surgical grade stainless steel		[183]
Bone graft implants	Bioactive glass-ceramic based on the CaO-SiO_2_-MgO-Na_2_O-Li_2_O system	Sinter-crystallization process	[184]
Antibacterial activity and enhanced bioactivity	Silver-doped wollastonite (CaSiO_3_) synthesized using natural waste		[96]
Implants	Hyroxyapatite surface layer		[185]
Apatite-wollastonite ceramics	Calcium hydroxyapatite and wollastonite powders	Compacted and sintered	[186]
Repair and replacement of living bone (load-bearing situations)	Wollastonite powder sprayed onto Ti–6Al–4V substrates	Plasma-sprayed wollastonite coatings	[187]

## Data Availability

All data are included within the article.
